# A Novel Tension Machine Promotes Bone Marrow Mesenchymal Stem Cell Osteoblastic and Fibroblastic Differentiation by Applying Cyclic Tension

**DOI:** 10.1155/2021/6647651

**Published:** 2021-08-05

**Authors:** Yi Zhao, Yiping Huang, Lingfei Jia, Ruoxi Wang, Kuang Tan, Weiran Li

**Affiliations:** ^1^Department of Orthodontics, Peking University School and Hospital of Stomatology, Beijing 100081, China; ^2^Department of Oral and Maxillofacial Surgery, Peking University School and Hospital of Stomatology, Beijing 100081, China; ^3^Central Laboratory, Peking University School and Hospital of Stomatology, Beijing 100081, China

## Abstract

Bone marrow mesenchymal stem cells (BMSCs) are intraosseous stem cells, and the effects of tensile strain on BMSC differentiation mediate several bone-related treatments. To study the response of BMSCs under tension, we designed and developed a small cellular tension instrument, iStrain. When iStrain applied tension on BMSCs, these cells exhibited convergence in the alignment direction and lengthening of the cell processes and cell body. Real-time quantitative polymerase chain reaction (RT-qPCR) and western blotting demonstrated that iStrain-mediated cyclic tension promotes the differentiation of BMSCs toward osteogenesis and fibrogenesis. And the mRNA and protein expression of differentiation-related genes changes with the extension of tension time.

## 1. Introduction

Stem cells, which are defined as cells with the capacity to self-proliferate and differentiate into new functional cells in tissue [1], are important in maintaining tissue integrity and normal function. Bone marrow mesenchymal stem cells (BMSCs) are a specific type of stem cell in bone with active self-renewing and multidifferentiation abilities [2]. These cells have the potential to develop into osteocytes, chondrocytes, adipocytes, and cells of other embryonic lineages [2].

Traditionally, stem cell differentiation is achieved by adopting cocktails of various growth factors. However, the linkage between differentiation and mechanical stimuli has gradually been revealed as mechanical stimulation has shown regulatory effects on cell proliferation, differentiation, apoptosis, and migration [3–7]. The main types of mechanical stimulation *in vivo* include shear stress, compressive stress, and tensile stress [6]. Among these types, the effects of tensile stress on BMSCs are clinically important. BMSCs are the main functional cells in bone distraction, as they can differentiate into osteoblasts and initiate osteogenesis. In the process of distraction osteogenesis, continuous tensile stress plays an important role in promoting BMSC proliferation and differentiation [8]. The osteogenic differentiation of BMSCs is also involved in bone regenerative activity in rapid maxillary expansion during orthodontic treatment [9].

To study the mechanoresponses of cells, researchers have developed many cellular mechanical loading models. Yang et al. classified them as substrate deformation-based approaches, weight approaches, hydrostatic approaches, centrifugation approaches, fluid flow approaches, vibration approaches, and 3-D loading models [10]. Among these, the substrate deformation-based mechanical loading model is the most widely used cellular mechanical loading device, especially for tension. These devices usually use an elastic membrane as the deformation substrate on which the cells are cultured. With the stretch of the elastic membrane, the cells will undergo tension [10].

**Figure 1:**
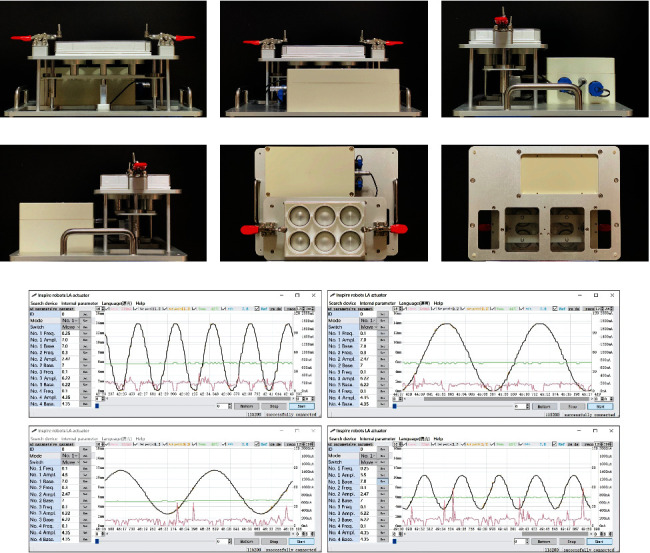
Images of the stretching device iStrain and its companion software. The front view (a), back view (b), side views (c, d), top view (e), and elevation view (f) of the stretching device iStrain. (g) Screenshots of the software showing different tension procedures.

However, available tension devices currently have a large size and strictly rely on an external power supply, both of which substantially constrict their applicable conditions and environment. To apply multiple strain loading modes in various circumstances, we designed a small stretching device named iStrain (Figures 1(a)–1(g)).

The objective of this study was to explore the response of BMSCs under different tension duration and the effects of tension on the osteoblastic and fibroblastic differentiation of BMSCs over time. To achieve this, we applied cyclic tensile strain to BMSCs using iStrain in vitro and observed the changes in cell morphology and internal structure, evaluated the survival and metabolic status of BMSCs by live/dead staining and Cell Counting Kit 8 (CCK-8) assays, and measured changes in mRNA and protein expression of differentiation-related genes by RT-qPCR and western blots.

## 2. Materials and Methods

### 2.1. Cell Culture

Human BMSCs were purchased from ScienCell (San Diego, CA, USA). Cells were used for experiments between the 3rd and 7th passages and cultured in alpha-modified Eagle's medium with regular glucose (ɑ-MEM; Gibco, Gaithersburg, MD, USA) supplemented with 10% fetal bovine serum (Gibco) and 1% penicillin-streptomycin solution (Gibco). Cells were incubated at 37°C in a humidified incubator containing 5% CO_2_.

### 2.2. Tensile Strain Experiments

BMSCs were seeded at a density of 3.0 × 10^5^ cells/cm^2^ on collagen I-coated 6-well BioFlex plates (Flexcell International, Burlington, NC, USA) and then incubated in ɑ-MEM until reaching 95% cell confluence. Subsequently, culture plates were subjected to cyclic tensile strain using iStrain with extension rates of 9%, 12%, and 15% at a frequency of 0.3 Hz for 12 h or subjected to cyclic tensile strain with a 12% extension rate at a frequency of 0.3 Hz for 6 h, 12 h, 24 h, 36 h, or 48 h. The BMSCs were then subjected to microscopic observation, immunohistochemistry staining, or RNA and protein extraction.

### 2.3. Live/Dead Staining

After exposure to cyclic tensile strain, BMSCs were stained with a live/dead staining kit (KGAF001, KeyGEN BioTECH Co Ltd., Nanjing, China). The culture medium was removed, and the BMSCs were washed three times with phosphate-buffered saline (PBS). Then, the BMSCs were stained in 2 ml of PBS supplemented with 0.4 *μ*M calcein AM (stained live cells) and 1.6 *μ*M propidium iodide (PI; stained dead cells) for 30 mins. The stained cells were observed using a fluorescence microscope (Nikon Eclipse 80i; Nikon Instech Co. Ltd., Kawasaki, Kanagawa, Japan). The live and dead cells exhibited green and red fluorescence, respectively.

### 2.4. CCK-8 Assays

CCK-8 assays were used to assess the proliferation of BMSCs treated with tensile strain. After exposure to cyclic tensile strain, BMSCs were seeded on 48-well plates. The medium was replaced every 2 days. 20 *μ*l of CCK-8 solution was added to 200 *μ*l of medium in one well of each group, and the cells were further cultured in a 5% CO_2_ incubator for 4 h. Then, 100 *μ*l of medium from each well was transferred to a new 96-well plate, and the absorbance at a wavelength of 450 nm was determined.

### 2.5. Cell Length Measurement

BMSCs were observed under a microscope, and cell length was measured. Six different positions of each well were selected, and six representative cells were taken at each position for measurement, and the results were obtained and analyzed.

### 2.6. Immunofluorescence Staining and Fluorescence Imaging

After application of tensile strain on BMSCs, BMSCs were fixed with 4% paraformaldehyde in PBS at room temperature for 15 min. After the cells were washed with PBS, the BMSCs were subsequently permeabilized with 0.1% Triton X-100 in PBS for 10 min. Then, the cells were washed with PBS again and blocked with 5% goat serum (ZLI-9022, Zhongshan Golden Bridge Biotechnology, Beijing, China) for 1 h. Then, the BMSCs were incubated with CytoPainter Phalloidin-iFluor 488 Reagent (Abcam, Cambridge, MA, USA) diluted at 1 : 1000 for 1 h at room temperature. Finally, the BMSCs were stained with DAPI (Solarbio Science & Technology Co., Ltd., Beijing, China) for 5 min at room temperature and were observed and photographed using a confocal system for imaging (LSM 5 EXCITER, Carl Zeiss, Jena, Germany).

### 2.7. RT-qPCR

Total RNA was extracted from BMSCs following treatment using TRIzol reagent (Invitrogen, Gaithersburg, MD, USA). A PrimeScript RT Reagent Kit (TaKaRa Biotechnology Co., Ltd., Dalian, China) was used to reverse transcribe cDNA from cellular RNA. RT-qPCR was performed using a Real-Time PCR Detection System (ViiA 7 Real-Time PCR System; Thermo Fisher Scientific, Wilmington, DE, USA) and FastStart Universal SYBR Green Master Mix (Roche Diagnostics GmbH, Mannheim, Germany) using the following parameters: 2 min at 50°C, 10 min at 95°C, and 40 cycles of 15 seconds at 95°C together with 1 min at 60°C. Glyceraldehyde 3-phosphate dehydrogenase (GAPDH) mRNA was used as the internal normalization control. The sequences of mRNA primers used in this study are listed in Table 1.

### 2.8. Western Blot Analysis

BMSCs were washed with ice-cold PBS and solubilized with RIPA lysis buffer containing 1% protease inhibitor cocktail (Solarbio Science & Technology Co.). Protein concentrations were measured by a BCA Protein Assay Kit (Thermo Fisher Scientific), and concentrations were adjusted to be the same. After 4× loading buffer was added, samples were heat denatured, and a total of 40 *μ*g of protein was used for western blot analysis. Prepared lysates containing equal amounts of protein were electrophoresed on a precast gel (Beyotime Institute of Biotechnology, Shanghai, China), and proteins were transferred to a polyvinylidene difluoride (PVDF) membrane. The transferred membranes were blocked with 5% BSA in TBST for 1 h at room temperature and were then incubated at 4°C overnight with anti-Runt-related transcription factor 2 (RUNX2) antibody; anti-osteopontin (OPN) antibody; anti-Collagen, type I, alpha 3 (COL-3) antibody; anti-Tenascin C (TN-C) antibody; anti-GAPDH antibody (Abcam); anti-Collagen, type I, alpha 1 (COL-1) antibody (Cell Signaling Technology, Danvers, MA, USA); or anti-Scleraxis (SCX) antibody (Santa Cruz Biotechnology, Dallas, TX, USA). After three washes with TBST, the membranes were incubated with anti-rabbit or anti-mouse secondary antibodies (ZB-2301 and ZB-2305, Zhongshan Golden Bridge Biotechnology, Beijing, China), which were diluted at 1 : 5000 at room temperature for 1 h. Chemiluminescence was produced using the Bio-Rad system, detected with a ChemiDoc MP Imaging System and analyzed using the ImageJ software.

### 2.9. Alizarin Red Staining

Alizarin red was used to detect calcium deposits formed by the BMSCs. After exposure to mechanical tension loading, BMSCs were reseeded in six-well plates. After 24 h of attachment, the medium was switched to ɑ-MEM containing 10% fetal bovine serum, 1% penicillin-streptomycin solution, 10 mM *β*-glycerophosphate, 100 nM dexamethasone, 200 mM vitamin C, and 2 mM KH_2_PO_4_. After 14 days of culture, the BMSCs were fixed in 4% paraformaldehyde at room temperature for 15 min and subsequently stained with Alizarin red (1% solution).

### 2.10. Statistical Analysis

All data are presented as the mean and standard deviation from three independent experiments. Differences among independent groups were analyzed by one-way analysis of variance (ANOVA) using IBM SPSS Statistics, version 26.0 (IBM Corp., Armonk, NY, USA). *P* < 0.05 was considered significant.

## 3. Device Design

The stretching device iStrain is a bioreactor invented to stimulate the tension state of cells from the muscle, lung, heart, blood vessel, skin, tendon, ligament, cartilage and bone *in vivo*. It is powered by a lithium battery and controlled by a computer program. The most obvious feature of iStrain is its compactness and battery power supply, which enable iStrain to adapt to a closed incubator with high humidity, suitable for cell culture to the maximum extent. Moreover, iStrain allows free programming to apply different force modes, including sine waves, triangular waves, and trapezoidal waves. The frequency and the specific duration of each action can also be adjusted on demand.

**Figure 2:**
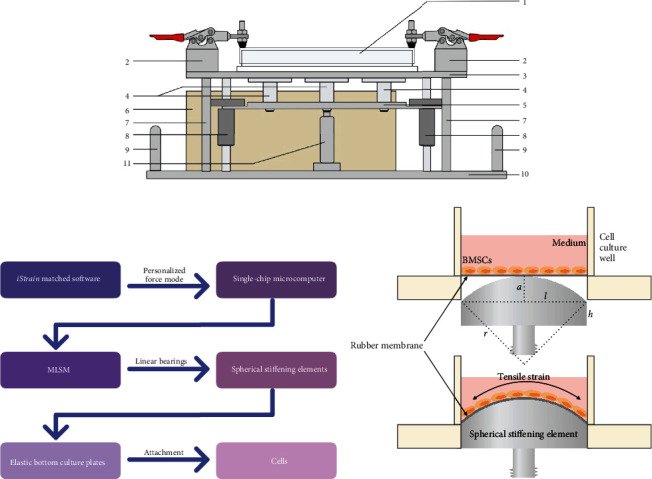
The structure and working principle of iStrain. (a) The structure of iStrain. 1: 6-well BioFlex plate; 2: culture plate fixtures; 3: culture plate support platform; 4: spherical stiffening elements; 5: movable platform; 6: power bank; 7: vertical support column; 8: linear bearings; 9: handles; 10: pedestal; 11: MLSM. (b) The workflow diagram of iStrain. A personalized force pattern is formed on iStrain companion software, and then, the force pattern is transferred to the microcomputer. After start-up, the microcomputer sends commands to the MLSM, which moves linearly according to the preset program. With the cooperation of two linear bearings, the MLSM drives the movable platform together with the spherical stiffening elements above it to move vertically, thus applying force to the elastic bottom of the culture plate and stretching the cells cultured on it. (c) The structure of the spherical stiffening element depends on five variables: *h*, *l*, *ε*, *r*, and *a*. *h* is the distance from the rubber membrane to the lowest bottom of the culture plate. *l* is the radius of the rubber membrane. For the BioFlex plates, *h* = 5 mm and *l* = 16.5 mm. *ε* refers to the extension rate, which is determined for each independent experiment. *r* refers to the radius of the spherical part, and *a* refers to its height. *r* and *a* are calculated as follows: *ε*% = [(*r*∙arcsin(*l*/*r*))/*l* − 1] × 100%; *a* = *r* − √(*r*^2^ − *l*^2^).

iStrain is composed of a single-chip microcomputer (inside the power bank; Figure 2(a), 6), a mechanical structure part and a power supply part. By using the visualized iStrain software on laptops, a personalized force mode can be easily set and switched. Then, through the USB port, the personalized force mode is transferred to the microcomputer inside the iStrain. After starting up, the microcomputer sends out instructions to the mechanical structure part. The mechanical structure part consists of the microlinear servo motor (MLSM; Figure 2(a), 11), the moving platform (Figure 2(a), 5) and the spherical stiffening elements (Figure 2(a), 4). When the instruction is acquired, the MLSM carries out linear motion according to the program set up in advance. With the cooperation of two linear bearings (Figure 2(a), 8), the MLSM drives the moving platform above it to move vertically. The spherical stiffening elements are fixed to the moving platform and are vertically below the elastic membranes of the cell culture plate. Thus, the vertical movement of the elements causes elastic deformation of the membranes and imposes regular stretching on the cells cultured on them (Figures 2(b) and 2(c)). The power supply part is composed of a lithium battery and a control switch. Both the single-chip microcomputer and the power supply part are packaged in one box (power bank, Figure 2(a), 6). The working time can reach 48 hours on a single charge.

For the maximum stretching effect of the equalized elastic membrane, the contact interface of the spherical stiffening element is designed as a spherical crown with low friction. The size of the element exactly fits the diameter of the cell culture well. When the element moves upwards, the elastic membrane will be deformed until the whole elastic membrane is pressed close to the spherical crown interface, making cells at all locations strained. A maximum of six spherical stiffening elements can be installed at the same time. By using different sizes of spherical stiffening elements, a 0-57% extension rate can be applied to the cells, and the precision of it can reach 0.01%.

With a size of 500 mm × 400 mm × 120 mm and a weight of 4900 g, the iStrain can be easily accommodated by regular incubators in a laboratory. All parts of the invention are moisture proof, and thus, the device can be used in a high-humidity environment and placed in a closed container as a whole without wiring.

## 4. Results

### 4.1. Cell Viability of BMSCs under Cyclic Tension

**Figure 3:**
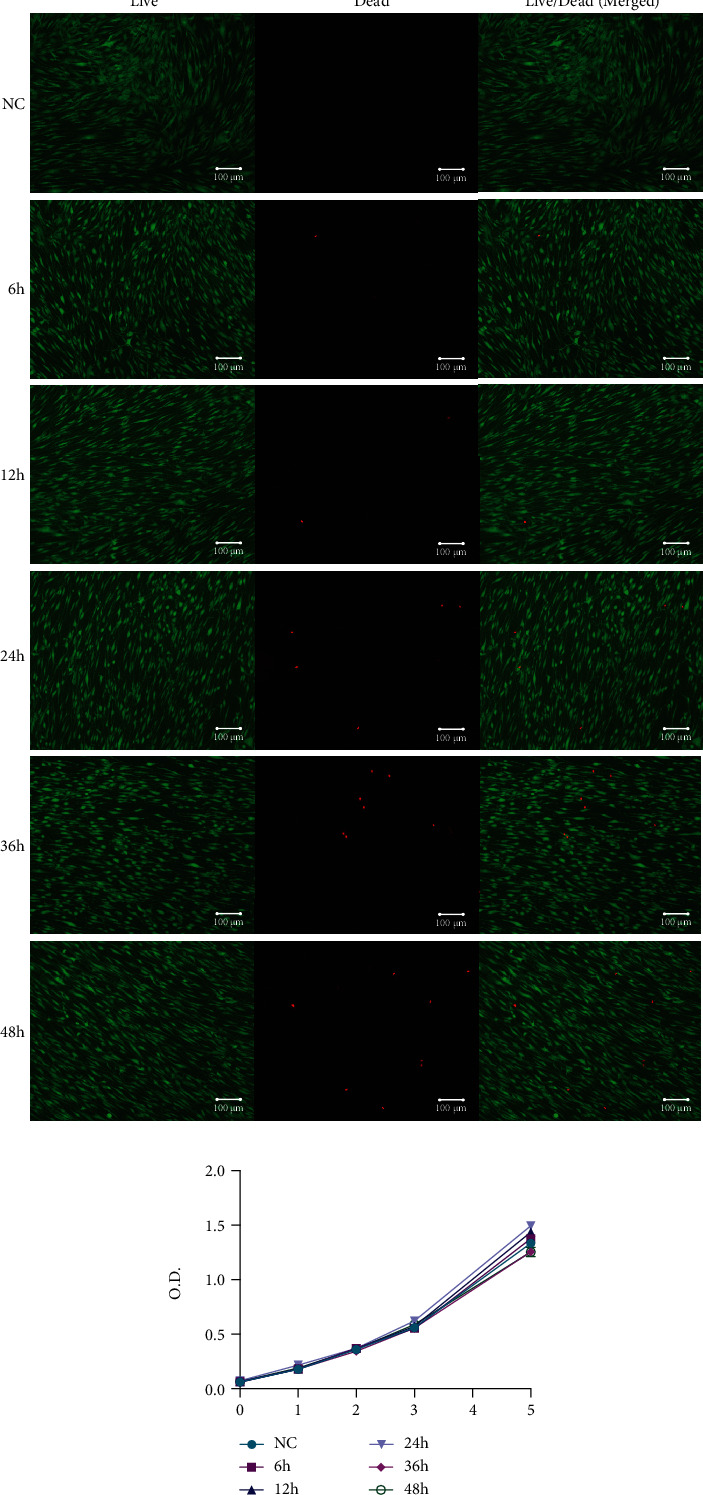
Cell viability of BMSCs under cyclic tension. (a) Live/dead staining of the negative control group and cyclic tension group at 6 h, 12 h, 24 h, 36 h, and 48 h. Live cells are stained with green fluorescence. Dead cells are stained with red fluorescence. (b) CCK-8 assay results show the effect of tensile strain lasting different times on cell proliferation of BMSCs. Different icons represent the negative control group and different time groups of 6 h, 12 h, 24 h, 36 h, and 48 h.

By using iStrain, we applied cyclic tensile strain to BMSCs at a frequency of 0.3 Hz for 6 h, 12 h, 24 h, 36 h, or 48 h with a 12% extension rate and investigated the damage of tensile strain to the cell viability of BMSCs utilizing live/dead staining and CCK-8 assays. Live/dead staining revealed that the number of dead cells increased with the extension of time from 0 h (negative control) to 48 h, and there was a remarkable increase between 12 h and 24 h, indicating significantly increased cell damage between 12 h and 24 h (Figure 3(a)). According to the CCK-8 assay results, there was no significant difference in the first three days between the different time groups. On day 5, there was a slight increase in the 6 h, 12 h, and 24 h time groups and a slight decrease in the 36 h and 48 h time groups compared with the control group (Figure 3(b)). Based on this, we hypothesized that a short period of tension would not have a significant negative effect on cell viability and proliferation, but extending the tension time to 24 h would affect cell viability, and continuing to extend the tension time to 36 h would affect cell viability and cell proliferation.

### 4.2. Cyclic Tension from iStrain Influences BMSC Morphology and Actin Filament Arrangement

**Figure 4:**
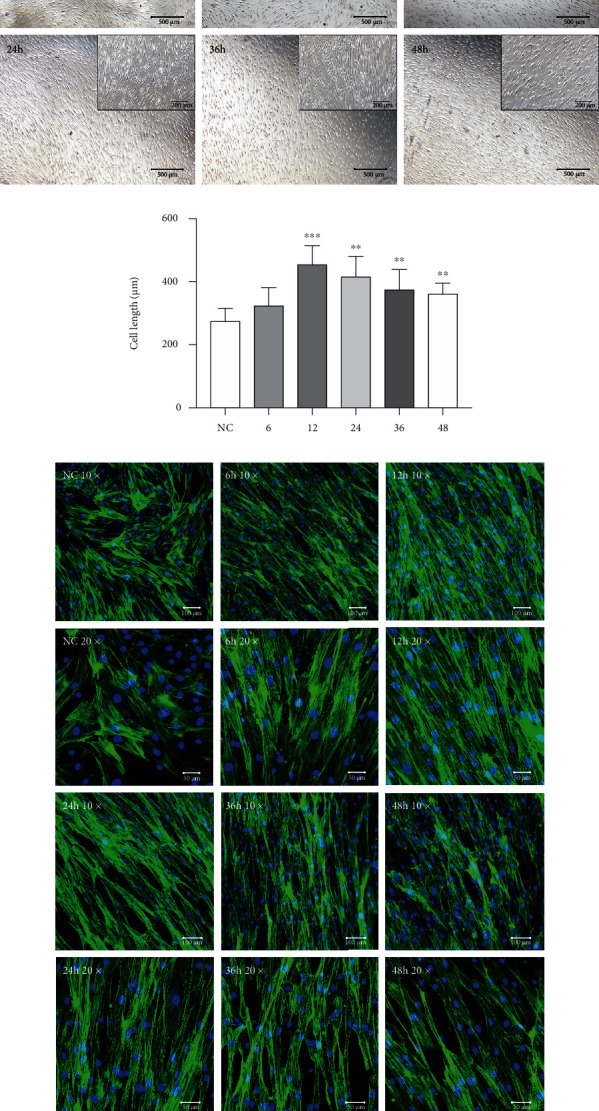
Cyclic tension influences BMSC arrangement and morphology. (a) Morphology and arrangement of BMSCs observed under the 4x and 10x microscopes after 6 h, 12 h, 24 h, 36 h, and 48 h of cyclic tension compared with those of the control group (without loading). Scale bars: 500 *μ*m/200 *μ*m. (b) Cell length of BMSCs measured under the microscope after 6 h, 12 h, 24 h, 36 h, and 48 h of cyclic tension compared with that of the control group. ^∗∗^*P* < 0.01 and ^∗∗∗^*P* < 0.001. (c) The green fluorescence indicates the F-actin. The blue fluorescence indicates the cell nucleus. Representative photographs of the control and cyclic tension groups at 6 h, 12 h, 24 h, 36 h, and 48 h under 10x and 20x microscopy are shown. Scale bars: 100 *μ*m/50 *μ*m.

By using iStrain, we applied cyclic tensile strain to BMSCs at a frequency of 0.3 Hz for 6 h, 12 h, 24 h, 36 h, and 48 h with a 12% extension rate. Under the microscope, the unstimulated control BMSCs were randomly arranged, whereas with the extension of the application time, the BMSCs exposed to the cyclic tensile strain were gradually arranged unidirectionally, perpendicular to the direction of the tensile force, accompanied by elongation of the cell processes and cell body. When the tension time exceeded 24 h, the intercellular space was gradually increased, and the cells were sparser (Figure 4(a)). By measuring the cell length under the microscope, we found that cell length gradually increased with extension of tension time and reached a peak at 12 h, followed by a decrease in cell length from 24 h to 48 h. Nevertheless, the cell lengths of the 24 h, 36 h, and 48 h groups were still longer than those of the negative control group (Figure 4(b)). These morphological and alignment changes were also confirmed by the arrangement of actin filaments. Immunohistochemistry staining demonstrated that actin filaments were arranged parallel in BMSCs treated with cyclic tensile strain but randomly oriented in the control cells (Figure 4(c)). The elongation of the cell processes and cell bodies was also observed with immunohistochemical staining of actin filaments.

### 4.3. Cyclic Tension from iStrain Influences the Expression of Osteoblastic Differentiation-Related Genes in BMSCs

**Figure 5:**
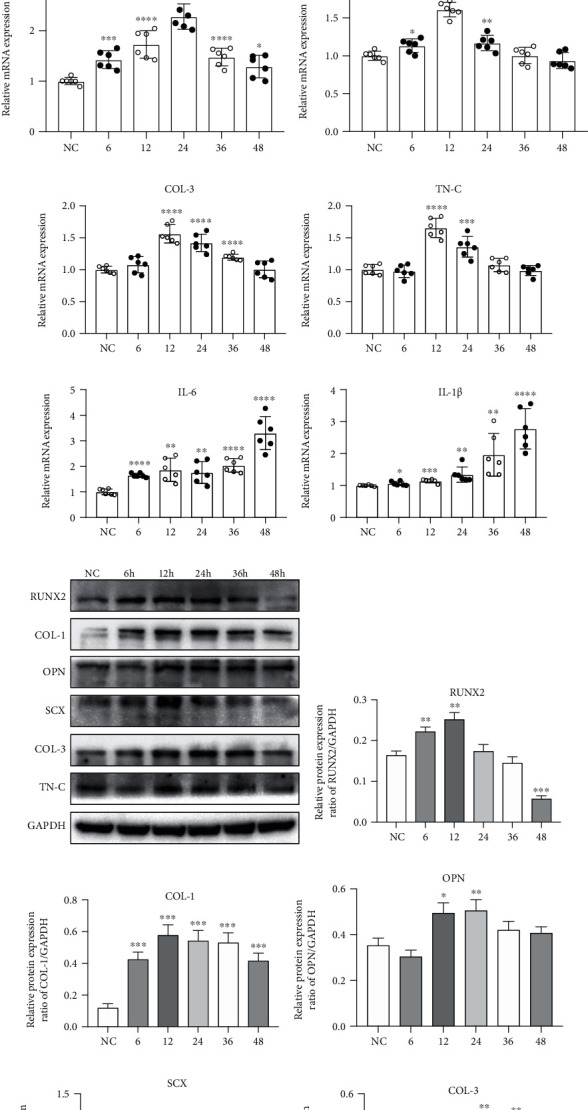
Cyclic tension promotes BMSC osteoblastic and fibroblastic differentiation. The mRNA expression levels of RUNX2 (a) and OCN (b) in response to cyclic tension force under different tension force values (including the control or with extension rates of 9%, 12%, and 15%) for 12 h. The mRNA expression levels of RUNX2 (c), COL-1 (d), OPN (e), SCX (f), COL-3 (g), TN-C (h), IL-6 (i), and IL-1*β* (j) in response to cyclic tension force with a 12% extension rate for the control or 6 h, 12 h, 24 h, 36 h, or 48 h of loading. Gene expression was calibrated using the GAPDH housekeeping gene. Data represent the mean ± SD; ^∗^*P* < 0.05, ^∗∗^*P* < 0.01, ^∗∗∗^*P* < 0.001, and ^∗∗∗∗^*P* < 0.0001 vs. the control. (h) Representative chemiluminescent images of western blot analysis of RUNX2, COL-1, OPN, SCX, COL-3, TN-C, and GAPDH in BMSCs. (l-q) Western blot analysis of RUNX2 (l), COL-1 (m), OPN (n), SCX (o), COL-3 (p), and TN-C (q). Gray value was calibrated using GAPDH. Data represent the mean ± SD; ^∗^*P* < 0.05, ^∗∗^*P* < 0.01, ^∗∗∗^*P* < 0.001, and ^∗∗∗∗^*P* < 0.0001 vs. the control.

Figures 5(a) and 5(b) show the changes in the mRNA expression of osteoblastic differentiation-related genes in BMSCs under different force values of cyclic tension for 12 h. The expression of RUNX2 and osteocalcin (OCN) was increased in the tension groups and peaked when the extension rate was 12%. Hence, we chose an elongation of 12% for the subsequent tension experiments.

Figures 5(c)–5(e) show the time course of mRNA expression of osteoblastic differentiation-related genes in BMSCs in response to cyclic tension force. Compared with that of the control group, the mRNA expression of RUNX2, COL-1, and OPN mRNA, detected by qPCR, gradually peaked at 12 h and 24 h with mechanical tension loading and then decreased thereafter.

### 4.4. Cyclic Tension from iStrain Influences the Expression of Fibroblastic Differentiation-Related Genes in BMSCs

Figures 5(f)–5(h) show the time course of mRNA expression of fibroblastic differentiation-related genes in BMSCs in response to cyclic tension force. Compared with that of the control group, the mRNA expression of SCX, COL-3, and TN-C mRNA gradually peaked at 12 h, which was followed by a gradual decrease.

### 4.5. Cyclic Tension from iStrain Influences the Expression of Inflammatory Genes in BMSCs

Figures 5(i) and 5(j) show the time course of changes in the mRNA expression of inflammation-related genes in BMSCs in response to cyclic tension forces. IL-6 and IL-1*β* mRNA expression, compared to controls, was upregulated in a time-dependent manner in the mechanically stimulated groups.

### 4.6. Cyclic Tension from iStrain Influences the Protein Expression of Osteoblastic and Fibroblastic Differentiation-Related Genes in BMSCs

Figures 5(k)–5(q) demonstrate the changes in the expression of osteogenic-related proteins and fibroblastic differentiation-related proteins in BMSCs in response to cyclic tension force.  Figure 5(k) shows the representative western blot results of RUNX2, COL-1, OPN, SCX, COL-3, TN-C, and GAPDH. Compared with that of the control group, the protein expression of RUNX2, COL-1, and OPN gradually peaked at 12 h and 24 h of mechanical tension loading and then decreased thereafter (Figures 5(l)–5(n)). The protein expression of SCX, COL-3, and TN-C also gradually peaked at 12 h and 24 h of mechanical tension loading and decreased thereafter (Figures 5(o)–5(q)).

### 4.7. Cyclic Tension from iStrain Influences the Mineralization of BMSCs

**Figure 6:**
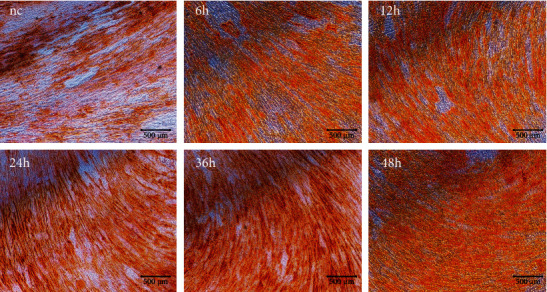
Cyclic tension promotes BMSC mineralization. The Alizarin red staining result of BMSCs in the control group and mechanically stimulated groups of 6 h, 12 h, 24 h, 36 h, and 48 h. Scale bars: 500 *μ*m.

Figure 6 shows the Alizarin red staining result of BMSCs in the different time groups. Alizarin red staining revealed a time-dependent increase in the color intensity, which suggests the mineralization ability increasing in BMSCs with the extension of tension time.

## 5. Discussion

In this research, to study the effects of tensile strain stimuli on BMSCs, we developed a small-scale program-controlled mechanical tension device, iStrain. By using this device, we first investigated the suitable extension rate for BMSCs. According to similar preliminary experimental research methods, the elongation rate of the BMSC tension assay was mostly set to vary from 10% to 15% [11, 12]. Considering that the force application method of iStrain may be different from that used by other researchers, we first set the extension rate to three values: 9%, 12%, and 15%. We examined the mRNA expression of RUNX2 and OCN in each group by RT-qPCR and found that the expression of RUNX2 and OCN increased in the tensile groups, and the peak appeared in the group with a 12% extension rate. Therefore, we chose the 12% extension rate for subsequent experiments, including microscope observation, immunohistochemistry staining, RNA and protein extraction, and Alizarin red staining. Then, we utilized RT-qPCR and western blotting to analyze the mRNA and protein expression of osteogenic differentiation- and fibroblastic differentiation-related genes. Cyclic tensile strain stimuli will promote the osteogenic and fibroblastic differentiation of BMSCs, as shown by the increased expression of RUNX2, COL-1, OPN, SCX, COL-3, and TN-C. In the mechanical tension-stimulated groups, the mRNA and protein expression of most genes peaked when the tension duration was between 12 h and 24 h rather than longer time point. There are two possible factors to explain this. First, cell viability and proliferation will be harmed by long-term tensile strain, as we noted before. Second, with the extension of time, the inflammatory reactions in BMSCs may be intensified, which will be harmful for osteoblastic and fibroblastic differentiation of BMSCs. The harmful effect of long-time cyclic tension on cell viability and proliferation can also explain the reason of the phenomena of increased intercellular space and sparse BMSC arrangement when the tension time exceeds 24 h, as less cell renewal and more cell death under long-time cyclic tension. The reason of why there are slight differences of mRNA and protein expression peak between different genes is correlated with different timing of the appearance of relevant markers. For example, compared with OPN, RUNX2 is a relative early marker of the osteogenic process. The mRNA and protein expression of RUNX2 peaked at 12 h, suggesting that early osteogenic differentiation activity is more active when the tension time is closed to 12 h, while the mRNA and protein expression of OPN peaked at 24 h, suggesting that late osteogenic differentiation activity is more active when the tension time is closed to 24 h.

When using iStrain for force application, BMSCs seeded on the elastic membrane will be strained together with the membrane. According to the manufacturer's protocols, the material of the bottom of BioFlex® culture plates is a type of flexible silicone elastomer, which is linear elastic isotropic. When we used iStrain to stretch the membrane, the deformation of the membrane is always in the range of elastic deformation. Plus, the surface of the spherical stiffening elements is designed as frictionless and stainless to minimize the friction between the elements and the membrane.

**Figure 7:**
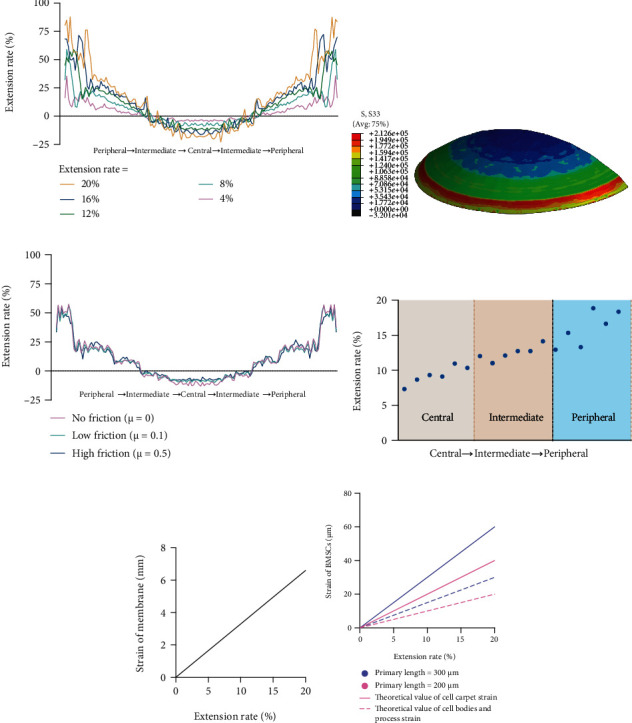
Strain of the membrane and BMSCs. (a) The strain on the membrane with different extension rates based on the three-dimensional finite element analysis. No friction between the membrane and the spherical stiffening elements supposed. The strain is determined as the ratio of the distances between two points under the stretched and unstretched conditions. (b) The three-dimensional rendering model of the distribution of tension force. (c) The three-dimensional finite element analysis of strain on the membrane under different frictions with 12% extension rate. (d) The distribution of strain from the central area to the peripheral area of the membrane. (e) The strain of the membrane equals extension rate times the primary length of the elastic membrane (2*l* = 33 mm). (f) The theoretical value of cell carpet strain is equal to the primary length times extension rate. The theoretical value of cell bodies and process strain is equal to half of the primary length times extension rate.

We also analyzed the strain of the membrane using three-dimensional finite element analysis by ANSYS® Software, version 19.0 (ANSYS Inc., Canonsburg, PA, USA). In the situation where friction was negligible, we compared the strain of the membrane with different extension rates. The results showed that the strain was closed to the theoretical extension rate in the intermediate area while smaller in the central area and larger in the peripheral area. The difference of the strain between different locations was smaller when the extension rate was relatively small and became larger when the extension rate increased (Figure 7(a)). The distribution of tension force was represented by different colors in the three-dimensional rendering model based on the three-dimensional finite element analysis, demonstrating an increasing trend from the central to the peripheral area (Figure 7(b)). And then, we compared the strain of the membrane under different frictions with 12% extension rate. The result showed that the strain has no significant changes between different friction groups (Figure 7(c)). Moreover, we also examined the strain of the membrane at different locations with 12% theoretical extension rate using flour particles as Wang et al. applied [13]. The extension rate was closed to 12% in the intermediate area while smaller in the central area and larger in the peripheral area, but the difference between them was considered to be acceptable (Figure 7(d)).

To sum up, the cells in the center of the membrane were deformed less than the ones next to the edges, once they were deformed spherically. The curve of the extension rate rose slowly from the center to the peripheral area and then ascended steeply in the most peripheral area. So, in our opinion, cell deformation in most part of the membrane could be considered uniform, except for the most peripheral area. And the experimental value of the extension rate was close to the theoretical one. However, the degree of the deviation from uniform stretching increased with the extension rate. Once the extension rate exceeded 16%, the nonuniformity of strain was thought to become unacceptable to be neglected.

The strain of the elastic membrane is equal to the primary length of the membrane times the extension rate, which can be written as the strain of the elastic membrane = 2*l* × extension rate (*l* is the radius of the membrane, which equals half of the primary length of the membrane) (Figure 7(e)). According to the research by Bieler et al., cell carpets show full strain transfer, while cell processes and cell bodies show about 50% strain transfer [14]. Therefore, the theoretical value of cell carpet strain is equal to the primary length times extension rate. The theoretical value of cell bodies and processes strain is equal to half of the primary length times extension rate (Figure 7(f)).

## Figures and Tables

**Table 1:** mRNA primer sequences used in this study.

Gene	Forward sequence (5′⟶3′)	Reverse sequence (5′⟶3′)
GAPDH	GCATTGCCCTCAACGACCACT	CCATGAGGTCCACCACCCTGT
RUNX2	ACTACCAGCCACCGAGACCA	ACTGCTTGCAGCCTTAAATGACTCT
OCN	AGCAAAGGTGCAGCCTTTGT	GCGCCTGGGTCTCTTCACT
COL-1	TGGACGCCATCAAGGTCTACTGC	GGAGGTCTTGGTGGTTTTGTATTCG
OPN	CGAGGTGATAGTGTGGTTTATGG	GCACCATTCAACTCCTCGCT
SCX	CAGCCCAAACAGATCTGCACCTT	CTGTCTTTCTGTCGCGGTCCTT
COL-3	TGGTCTGCAAGGAATGCCTGGA	TCTTTCCCTGGGACACCATCAG
TN-C	GTCACCGTGTCAACCTGATG	GTTAACGCCCTGACTGTGGT
IL-6	AGACAGCCACTCACCTCTTCAG	TTCTGCCAGTGCCTCTTTGCTG
IL-1*β*	TGGCAACTGTTCCTG	GGAAGCAGCCCTTCATCTTT

GAPDH: glyceraldehyde 3-phosphate dehydrogenase; RUNX2: Runt-related transcription factor 2; OCN: osteocalcin; COL-1: collagen, type I, alpha 1; OPN: osteopontin; SCX: Scleraxis; COL-3: collagen, type I, alpha 3; TN-C: Tenascin C.

## Data Availability

The data used to support the findings of this study are included within the article.
